# Dual activity of Minnelide chemosensitize basal/triple negative breast cancer stem cells and reprograms immunosuppressive tumor microenvironment

**DOI:** 10.21203/rs.3.rs-3959342/v1

**Published:** 2024-02-22

**Authors:** Hasan Korkaya, Fulya Koksalar Alkan, Ahmet Caglayan, Hilmi Alkan, Elayne Benson, Yunus Gunduz, Ozge Sensoy, Serdar Durdagi, Elbrus Zarbaliyev, Gregory Dyson, Hadeel Assad, Austin Shull, Ahmed Chadli, Huidong Shi, Gurkan Ozturk

**Affiliations:** Wayne State University School of Medicine; Presbyterian College; Augusta University; Augusta University

## Abstract

Triple negative breast cancer (TNBC) subtype is characterized with higher EMT/stemness properties and immune suppressive tumor microenvironment (TME). Women with advanced TNBC exhibit aggressive disease and have limited treatment options. Although immune suppressive TME is implicated in driving aggressive properties of basal/TNBC subtype and therapy resistance, effectively targeting it remains a challenge. Minnelide, a prodrug of triptolide currently being tested in clinical trials, has shown anti-tumorigenic activity in multiple malignancies via targeting super enhancers, Myc and anti-apoptotic pathways such as HSP70. Distinct super-enhancer landscape drives cancer stem cells (CSC) in TNBC subtype while inducing immune suppressive TME. We show that Minnelide selectively targets CSCs in human and murine TNBC cell lines compared to cell lines of luminal subtype by targeting Myc and HSP70. Minnelide in combination with cyclophosphamide significantly reduces the tumor growth and eliminates metastasis by reprogramming the tumor microenvironment and enhancing cytotoxic T cell infiltration in 4T1 tumor-bearing mice. Resection of residual tumors following the combination treatment leads to complete eradication of disseminated tumor cells as all mice are free of local and distant recurrences. All control mice showed recurrences within 3 weeks of post-resection while single Minnelide treatment delayed recurrence and one mouse was free of tumor. We provide evidence that Minnelide targets tumor intrinsic pathways and reprograms the immune suppressive microenvironment. Our studies also suggest that Minnelide in combination with cyclophosphamide may lead to durable responses in patients with basal/TNBC subtype warranting its clinical investigation.

## Introduction

Among American women, breast cancer is the most common malignancy, after skin cancer. Metastatic breast cancer is the leading cause of cancer-related death among women. Women with the basal/TNBC subtype constitute 15–20% of breast cancer patients and are often diagnosed with aggressive/metastatic disease^1^. Basal/TNBC tumors exhibit an epithelial mesenchymal transition (EMT) phenotype and cancer stem cell (CSC) properties^2^, which contribute to metastasis and treatment resistance^3–5^. In addition, the immunosuppressive tumor microenvironment (TME) in TNBC has been implicated in the failure to respond to conventional and targeted therapies^6^. Thus, an improved understanding of the mechanism by which immunosuppressive TME contributes to therapy resistance will lead to the development of alternative therapeutics. Minnelide, a prodrug of triptolide, has shown anti-tumorigenic activities in multiple malignancies by targeting super-enhancers (SE) and anti-apoptotic pathways, such as heat shock protein 70 (HSP70) signaling^7 8^. This small-molecule inhibitor covalently bound to the XPB subunit of transcription factor II H (TFIIH) super-enhancer complex regulates many targets, including *c-Myc*^9^. Genomics studies have revealed a distinct SE landscape in basal/TNBC compared with other subtypes of breast cancer^10^. Consistent with this notion, transcriptional SEs have been implicated in cancer stemness programs for number of malignancies, including breast cancer^11^. Minnelide targets CD133 + tumor initiating cells in syngeneic models of pancreatic ductal adenocarcinoma^12^. Other modes of anti-tumorigenic activities of Minnelide include targeting HSP70 signaling has been shown to be a major target in multiple malignancies^13^. We previously reported that the TNFAIP3(A20)/HSP70 signaling axis is selectively upregulated in TNBCs in response to cytotoxic agents^14^ suggesting the therapeutic utility of Minnelide for this subtype. However, the antitumorigenic activity of triptolide has also been reported in luminal breast cancer subtype via different mechanisms, such as downregulation of ERα signaling and lysosome-mediated cell death^15^.

In addition to their tumor intrinsic role, epigenetic regulators are implicated in the reprogramming of stromal/immune cells within the tumor microenvironment^16^ and several inhibitors of epigenetic modifying enzymes are being tested in preclinical and early phase clinical trials to improve the efficacy of immune checkpoint inhibitors (ICI) in solid tumors^17^. Super-enhancers have been shown to drive immune evasion by controlling the expression of genes such as PD-L1/PD-L2 and TGFβ which are critical players in immune suppression^18^. Consistent with this notion, triptolide effectively suppressed IFN-γ-driven PD-L1 expression in breast cancer cell lines^19^. Minnelide in combination with low-dose gemcitabine and paclitaxel exhibited enhanced therapeutic activity by reducing stromal collagen content in pancreatic cancer^20^. Furthermore, embryonic reprogramming transcription factors such as Oct4 and Sox2 induce a bromodomain (BRD)-dependent immunosuppressive microenvironment in glioblastoma stem-like cells^21^. Overall, studies thus far suggest a wide range of anti-tumorigenic activities of Minnelide in preclinical studies of multiple malignancies^22^.

Given its promising preclinical activity^23^, Minnelide is now currently being evaluated in Phase II clinical trials (NCT04896073) in patients with advanced refractory pancreatic carcinoma^24^. At least 16 weeks of stable disease as an endpoint was intended as per the response evaluation criteria in solid tumors (RESIST). Scientific exploratory end points include evaluation of *Myc* expression and accessibility of loci for *Myc* gene in pre- and post-treatment tumors and profiling of circulating immune cell populations in patients’ blood^24^.

We therefore reasoned that Minnelide may exhibit dual activity by targeting tumor intrinsic pathways and reprogramming the immune microenvironment in the TNBC subtype, which is characterized by an immunosuppressive TME^6^. Our studies provide evidence that Minnelide targets CSCs by inhibiting multiple tumor intrinsic signaling pathways such as A20/HSP70, Myc, Vimentin and BRD4, which have been widely implicated in driving aggressive properties of the TNBC subtype. Minnelide sensitizes tumors to cyclophosphamide (CTX) in syngeneic mice, eradicating disseminated tumor cells following resection of residual tumors. This may be due to the reprogramming of the tumor microenvironment, which leads to enhanced cytotoxic T cell infiltration and improved overall outcomes.

## Materials and Methods

### Cell lines and reagents

4T1, AT-3, E0771, EMT6, J774A.1, Raw264.7, MCF7, MDA-MB-231, Sum-159 and ZR-75–1cell lines were purchased from American Type Culture Collection (ATCC). All cell lines were tested for mycoplasma contamination by PCR analyses using a Universal Mycoplasma Detection Kit (30–1012K^™^, ATCC). The 4T1 cell line is infected with luciferase-expressing lentivirus, and stable cell lines were generated to monitor tumor growth in live animals and tissues. 4T1, 4T1-Luc, EMT6, MCF-7 and ZR-75–1 cell lines were maintained in RPMI1640 supplemented with 10% fetal bovine serum, and antibiotic/antimycotic 10,000U/ml. E0771 cell line was maintained in DMEM supplemented with 10% fetal bovine serum, and antibiotic/antimycotic 10,000U/ml. The MDA-MB-231 cell line was maintained in DMEM supplemented with 5% fetal bovine serum, and antibiotic/antimycotic 10,000U/ml. Sum-159 cells were maintained in Ham’s F12 supplemented with 5% fetal bovine serum, 5mg/ml insulin, 1mg/ml hydrocortisone, and antibiotic/antimycotic 10,000U/ml). Raw264.7 cell line was maintained in DMEM supplemented with 10% fetal bovine serum.

Minnelide (HY-124584, MCE) was resuspended in DMSO for *in vitro* assays and 10% DMSO in 90% (20% SBE-β-CD (HY-17031, MCE)) in saline to obtain a clear solution, as described by the manufacturer. Vehicle controls were used at corresponding concentrations for each experiment. Cyclophosphamide (CTX) was resuspended in saline. Recombinant mouse IL-4 (404-ML/CF, R&D Systems) and recombinant human TGF-beta1 (240-B-010/CF, R&D Systems) were used at the indicated concentrations. LPS was purchased from Sigma-Aldrich and resuspended in cell culture medium to induce macrophage differentiation.

### In vitro studies

A cell proliferation assay from Promega (G3580) was used to assess the cytotoxicity and proliferation of cells in the presence of Minnelide. Briefly, 1,000 cells were seeded in a 96-well plate and treated with increased doses of Minnelide the next day. A set of wells were maintained with medium only for background subtraction. 20μl of MTS solution were added to each well and incubated for 2 h at 37°C and the absorbance was recorded at 490nm. Experiment was repeated 2 independent times in triplicates.

Flow cytometry was performed using Annexin V Binding buffer (422201, BioLegend). Briefly, cells were treated with Minnelide for 48 h. After collecting the cells, 100,000 cells were resuspended in Annexin V Binding buffer and stained with fluorochrome conjugated Annexin V antibody (640920, 640945, BioLegend) and 7-AAD (420404, BioLegend) or DAPI (564907, BD Biosciences). Murine mammary cancer stem cells were analyzed by flow cytometry using CD24(138504 BioLegend, 1:100), CD29 (102216, BioLegend, 1:100). The samples were analyzed using a NovoCyte Quanteon Flow Cytometer CyTEK Northern Lights Full Spectrum cytometer. Experiments were repeated 3 independent times.

Immune profiling assays of monocyte and macrophage cell lines were performed using fluorescent conjugated antibodies CD11b (101243, BioLegend 1:250), CD11c (117353, BioLegend, 1:250), Ly6C (128018, BioLegend, 1:250), Ly6G (127624, BioLegend, 1:250), CD86 (105011, BioLegend, 1:250). The viability dye Zombie Aqua (423102, BioLegend, 1:500) were chosen according to antibody/fluorochrome compatibility of panel. The samples were analyzed using a NovoCyte Quanteon Flow Cytometer CyTEK Northern Lights Full Spectrum cytometer.

### Protein modeling, preparation, and receptor grid generation

The NBD domain of Hsc70 pertaining to Bos taurus (PDB ID: 3HSC) containing ADP nucleotide was present in the protein database (PDB), yet the structure of the whole protein was not available. The amino acid similarity between the NBD domains of Bos taurus Hsc70 and human Hsp70 proteins was 88.542%. Therefore, the full structure of the human Hsp70 was modeled using the crystal structure of the bacterial Hsp70, DnaK (PDB ID:2KHO) and NBD of Hsc70 belonging to Bos taurus using Swiss-Models program. The Protein preparation module in Maestro molecular modeling package (Maestro, 2018) was used for protein preparation. Crystal structure of human HSP70 complexed with VER-155008 was downloaded from protein data bank (PDB ID: 4IO8) and aligned with the modeled HSP70 3D structure. The co-crystallized ligand (VER-155008) is merged to the HSP70 and ligand-bound model protein structure is saved. The PROPKA^25^ (pH, 7) and OPLS3 force field^26^ were used for protonation states, structural optimization and minimization, respectively. Furthermore, receptor grid generation in Glide^3^ was used to generate the grid boxes at the active site of merged ligand.

### Ligand preparation

LigPrep module (Ligprep, 2018) of Maestro molecular modeling with the OPLS3 force field^26^ was used for preparation of ligands. Epik^27^ module in Ligprep was used to assign potential ionization states at pH 7. 3D geometry optimization and energy minimization were performed to generate the 3D structures of the ligands. OPLS3 forcefield^2^ was used for energy minimization, by choosing standard energy function and RMSD cut off of 0.01 Å for the generation of low energy conformations.

### Mouse tumor implantation

All animal procedures were performed in accordance with the Institutional Animal Care and Use Committee (IACUC) at Augusta University (AU). The animal protocol for the procedures conducted in this study was approved by the Laboratory of Animal Services (LAS) at AU. All mice were housed at room temperature with 50–70% humidity, and 12/12 hour light/dark cycle. Animals were housed as maximum as 5 mice per cage during the experiments. BALB/c female mice (5 weeks old) were purchased from The Jackson Laboratory. A total of 50,000 4T1-Luc cells were implanted into the 4th mammary fat pads of mice in a 50/50% media/Matrigel (Corning) mixture. For the *in vivo* studies, mice were treated with Minnelide (0.5mg/kg, daily, i.p.) and/or cyclophosphamide (Sigma Aldrich) (150mg/kg, weekly, i.p.). For the survival experiments, tumors were resected by the third week of 4T1-Luc mammary fat pat injection. Animals were followed up for primary tumor and/or metastatic growth by weekly bioluminescence imaging using AMI (Spectral Instruments Imaging), and images were analyzed using Aura software. Total number of 32 mice were used. 5 mice were used for control group and treated with vehicle. 5 mice were used for CTX and Minnelide groups. 6 mice were used for CTX and Minnelide combinational treatment group. For the resection experiments, 3 mice in control, 4 mice in Minnelide and 4 mice in CTX and Minnelide combination group were used. Minnelide treatment started on day 3 and CTX treatment started on day 7 after 4T1-Luc mammary fat pad injection. PBMCs from tail vein during the tumor growth and resected tumor and organs at the endpoint of each experiment were tested for immune cell markers as described in Flow Cytometry methods.

### Flow Cytometry

For immune profiling, single-cell suspensions were prepared from blood, spleen, lungs, and tumors. Lung and tumor tissues were dissociated and digested with collagenase/hyaluronidase (07912, STEMCELL Technologies, USA). The spleens were smashed on a cell strainer with a syringe plunger. For blood and each organ, red blood cells were lysed using 1X RBC Lysis Buffer (10X, 420302, BioLegend). Single cell suspensions has been maintained in 2% FBS-in PBS until staining. Flow cytometry based immune profiling of MDSCs and T cells was performed using fluorescent conjugated antibodies against CD45 (103155, BioLegend, 1:250) CD11b (101243, BioLegend 1:250), CD11c (117330, BioLegend, 1:250), Ly6C (128018, BioLegend, 1:250), Ly6G (127624, BioLegend, 1:250) and CD86 (105011, BioLegend, 1:250). The viability dyes Zombie Aqua (423102, Biolegend, 1:500) and Zombie Violet(423114, Biolegend, 1:500) were chosen according to the panels for each immune profiling assays. Samples were analyzed using a NovoCyte Quanteon Flow Cytometer and a CyTEK Northern Lights Full Spectrum Cytometer. Data analysis was performed using FlowJo v.10.

### RT-PCR and Western Blot analysis

Total RNA was extracted using the RNeasy Mini Kit (74536, Qiagen) and 500ng of RNA was used to make cDNA using the iScript cDNA Synthesis Kit (1708891, BioRad). For RT-PCR analysis, gene-specific primers ordered from KiCqStart SYBR Green Primers (Millipore Sigma) were used. Cebpd (F—5’-ATCACTTAAAGATGTTCCTGC − 3’, R—5’- TGTCTTCACTTTAATGCTCG − 3’), Ifnar1 (F—5’-CTAAGATAAGCATGGAGAAGG-3’, R—5’- AATCCAGATCGTGGAAAAAC-3’), Il1b (F—5’-GGATGATGATGATAACCTGC-3’, R—5’-CATGGAGAATATCACTTGTTGG-3’), Il4 (F—5’-CTGGATTCATCGATAAGCTG − 3’, R—5’- TTTGCATGATGCTCTTTAGG − 3’), Il6 (F—5’-AAGAAATGATGGATGCTACC-3’, R—5’-GAGTTTCTGTATCTCTCTGAAG-3), Il10 (F—5’-CAGGACTTTAAGGGTTACTTG − 3’, R—5’-ATTTTCACAGGGGAGAAATC − 3) and Tlr4 (F—5’-GATCAGAAACTCAGCAAAGTC − 3’, R—5’-TGTTTCAATTTCACACCTGG − 3’). Relative gene expression at the mRNA level was normalized against the internal control ACTB (F—5′-GATGTATGAAGGCTTTGGTC-3′, R—5′-TGTGCACTTTTATTGGTCTC-3′) gene (ΔCt = Ct (target gene) − Ct (internal control gene)). Relative fold change was measured using the 2^−ΔΔCt^ formula and compared with the control cells. Means and differences with 95% confidence intervals were obtained using GraphPad Prism 10 (GraphPad Software Inc.). Two-tailed Student’s t test was used for unpaired analysis to compare the average expression between conditions. Statistical significance was set at p < 0.05 were For Western Blot analysis, cells were lysed in Pierce RIPA Buffer (89901, 78440 Thermo Scientific). 20μg of each protein in 4X Laemmli Sample Buffer (1610747, Bio-Rad) was boiled at 95°C for 5 min and subjected to SDS-PAGE. Proteins were transferred to a polyvinylidene fluoride (PVDF) membrane (1620177, Bio-Rad) using a semi-dry Trans-Blot (Bio-Rad). Blots were first incubated in TBS blocking buffer containing 2% non-fat dry milk or 2% BSA (for phosphor-specific antibodies) for 1 h at room temperature and then incubated with the respective primary antibodies diluted in TBS-T (containing 0.1% Tween20 and 2% BSA) overnight at 4°C in the dark. Blots were washed and incubated with appropriate secondary antibodies in TBS-T and detected using Clarity Western ECL Substrate (1705061, Bio-Rad). Antibodies against cMyc (ab32072, Abcam), vimentin (5741, Cell Signaling Technology), A20/TNFAIP3 (NBP1–77533, Novus), HSP70/72(ADISPA-810-F, Enzo Life Sciences), Brd4 (ab128874, Abcam), and ß-actin (664803, BioLegend). All antibodies were used at 1:1000 dilution. Uncropped scans of the blots are provided in supplementary figures.

### Statistical Analysis.

The statistical analysis applied to each graph is indicated in the figure legends. Briefly, Unpaired two-tailed t-tests were applied to determine the significance between two treatment groups, and one-way analyses variance (ANOVA) was used for variance analysis between the control and every other group. In vitro experiments were repeated in three different time points and were indicated with the mean ± SD. For survival percentiles, the data were submitted to Kaplan-Meier curve tests and differences between two groups were submitted to the log-rank test. All statistical analyses were performed using GraphPad Prism (version 10).

## Results

### Minnelide suppresses cell proliferation in human and murine TNBC cell lines in a dose dependent manner

**Figure 1:**
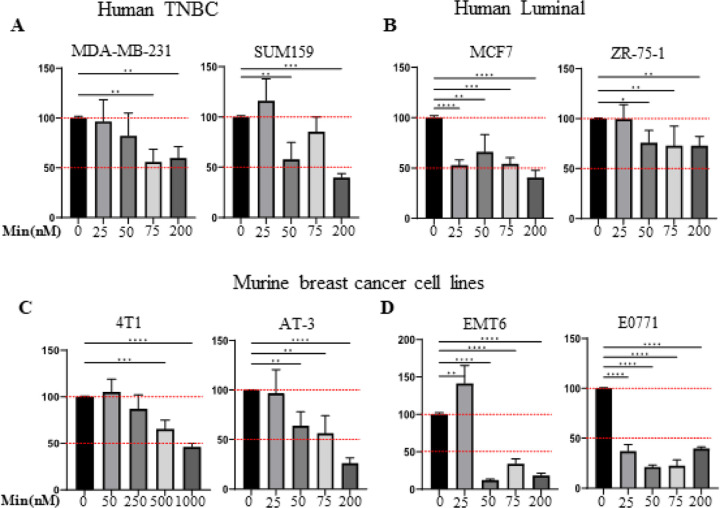
Minnelide activity on tumor cell proliferation of human and murine breast cancer cell lines: Human breast cancer cell lines, MDA-MB231, Sum159, ZR75–1, MCF7 AND murine breast cancer cell lines, EMT6, E00771, AT3 and 4T1 were incubated with indicated doses of Minnelide for 48 hours and cell viability was measured by MTT assay. **A-D.** Minnelide reduces proliferation in a dose dependent manner in TNBC lines and luminal subtype. Experiments were done 2 independent times in triplicate and cell viability was shown in percentages (± SD).One-Way ANOVA is used to compare test groups to control. *P < 0.05, **P < 0.01, ***P < 0.001, ****P < 0.0001.

We first assessed the effect of Minnelide on cell viability in multiple human and murine breast cancer cell lines representing TNBC or the luminal subtype. We treated human breast cancer cell lines MDA-MB231, Sum159, MCF7 and ZR-75–1) and murine breast cancer cell lines (4T1, AT3, EMT6 and E00771) with increasing doses of Minnelide ranging from 25nM to 1μM for 48 h and assessed cell viability using the CellTiter 96 Aqueous One Solution. Although Minnelide significantly reduced cell viability in all breast cancer cell lines in vitro (Fig. 1A–D), TNBC cell lines exhibited a dose-dependent reduction in viability upon treatment with increasing doses of Minnelide (Fig. 1A and C) with the exception that the 4T1 cell line required relatively higher doses of the drug (Fig. 1C, left panel). In contrast, luminal breast cancer cell lines appeared to show a non-specific toxicity in response to treatment (Fig. 1B and D). Brightfield images of the cell lines at the time of the viability assay support the dose-dependent activity of Minnelide on TNBC and non-TNBC cell lines (Supplementary Fig. S1A-D).

### Minnelide specifically targets CSC population in TNBC cell lines by inducing apoptotic cell death

**Figure 2:**
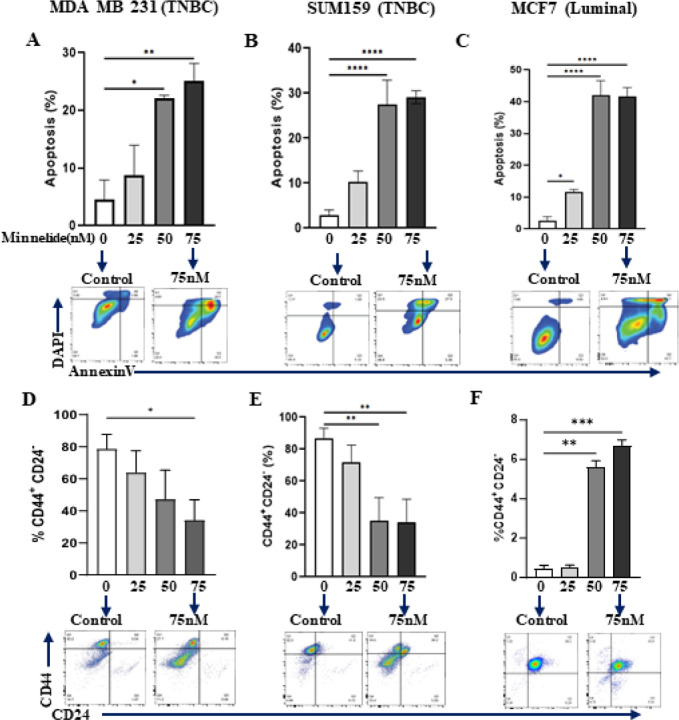
Minnelide significantly reduces CSC population in MDA-MB231 via inducing apoptotic cell death. Breast cancer cell lines, MDA-MB-231 and MCF7 were incubated with indicated doses of Minnelide for 48 hours and apoptotic cell death and CSC population was evaluated by flow cytometer. **A, B,** Minnelide induces a dose dependent cytotoxic cell death particularly in CSC **(d, e)** population of MDA-MB231 and Sum159 TNBC cell lines in vitro. **C, F,** Minnelide had no activity on the CSC population of luminal MCF7 cell lines while it induced a significant apoptotic cell death in these cells with increasing doses of Minnelide. Experiments were done in triplicates and values were shown in percentages (± SD). One-Way ANOVA is used to compare test groups to control. *P < 0.05, **P < 0.01, ***P < 0.001, ****P < 0.0001.

**Figure 3:**
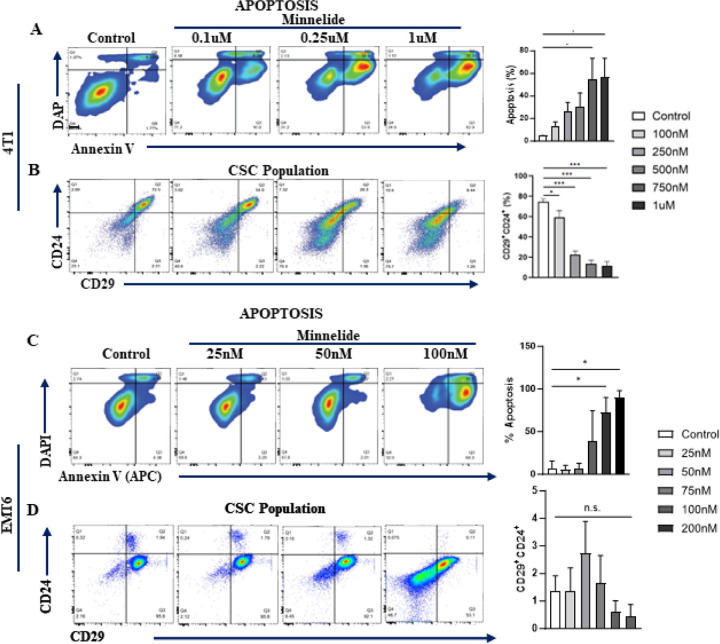
Minnelide targets CSC population in murine 4T1 tumor cells. Murine 4T1 and EMT6 tumor cells were incubated with indicated doses of Minnelide for 48 hours and apoptotic cell death and CSC population was evaluated by flow cytometer. **A, B.** Minnelide induces a dose dependent apoptotic cell death in 4T1 cells in vitro in addition to the reduced capacity of cancer stemness. **C, D.** Apoptotic cell death was increased by the increasing doses of minnelide while CSC population is not affected in vitro in EMT6 cells for 48 hours. Experiments were done in triplicates and values were shown in percentages (± SD). One-Way ANOVA is used to compare test groups to control. *P < 0.05, **P < 0.01, ***P < 0.001.

Given the distinct super-enhancer landscape in the human TNBC subtype^10^ as well as its role in reprogramming of the CSC phenotype^28^, we investigated whether Minnelide can specifically target the CSC population. MDA-MB231, SUM159 and MCF7 cell lines were treated with increasing doses of Minnelide for 48 h and subjected to flow cytometry analyses to evaluate apoptotic cell death. Minnelide treatment induced significant apoptotic cell death in all the three cell lines (Fig. 2A–C). Next, we examined the CSC population assessed suing the CD44^+^CD24^−^ phenotype^29^ in these cell lines after 72 h treatment with the indicated doses of Minnelide. There was a dose-dependent and significant reduction in the CSC population in both TNBC cell lines, MDA-MB231 and Sum159 (Fig. 2D and E). In contrast, the CSC population was unexpectedly increased in the luminal MCF7 cell line despite significant apoptotic cell death (Fig. 1F). We further evaluated the activity of Minnelide in murine breast cancer cell lines, 4T1 and EMT6. The murine 4T1 tumor model is a well-characterized representative of the human TNBC subtype^30^, which constitutes a high CSC population and exhibit aggressive/metastatic properties compared with the less invasive EMT6 tumor model^31 32^. As expected, Minnelide induced a dose-dependent induction of apoptotic cell death and concomitant reduction of the CSC population defined by CD24^+^CD29^+^ phenotype in 4T1 tumor cells after 48- and 72-hours treatment respectively (Fig. 3A and B). However, Minnelide induced nonspecific toxicity in EMT6 tumor cells as there was massive cell death at 75nM and higher doses while there was no activity with lower doses of the drug (Fig. 3C). Moreover, the CSC population in EMT6 cells was not significantly changed upon Minnelide treatment despite significant cell death (Fig. 3D). Together our *in vitro* data provide evidence of the specificity of Minnelide for the basal-like/TNBC subtype.

### Minnelide targets HSP70 and Myc pathways in basal/TNBC subtype

**Figure 4:**
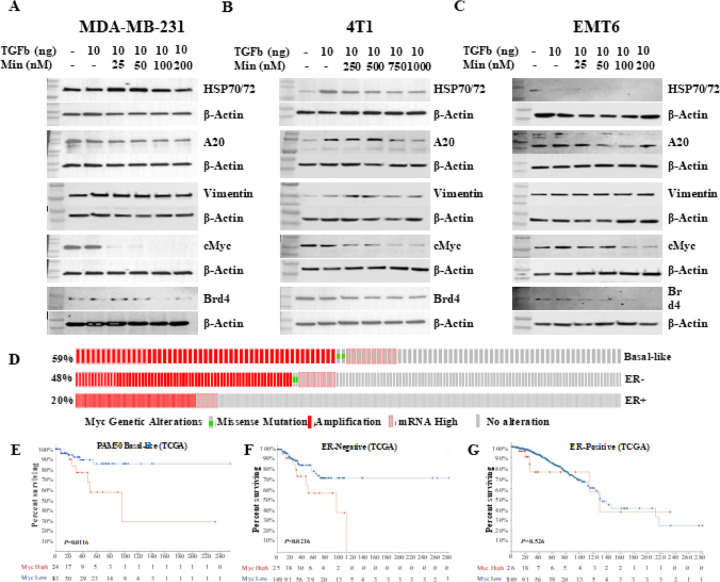
Minnelide suppresses TGFb-induced upregulation of signaling pathways. **A-C,**Protein expression of HSP70/72, A20, Vimentin, MYC and BRD4 was shown in human MDA-MB-231 and murine 4T1 and EMT6 cell lines treated with increased dose of Minnelide in the presence and absence of recombinant human TGFb. Experiments were done in duplicates. **D,** TCGA data showing the genetic alterations of *myc* in Basal-like, ER- and ER+ breast cancer subtypes. **E-G,** Kaplan-Meier TCGA data showing the association between the Myc expression levels and survival rates among the patients with high gene expression (red) in Basal-like, ER- and ER+ breast cancer compared to patients with low expression (blue), respectively.

The broad anti-tumorigenic activity of Minnelide has been attributed to its ability to target multiple signaling pathways^22^. Although it was shown to target XBP subunit of the transcription factor II H (TFIIH)-BRD4 super-enhancer complex, specific protein-ligand complex was not shown for HSP70 binding. We performed a molecular docking algorithm by using the Glide/XP program and determined the specific binding site of Minnelide on HSP70 (Supplemental Fig. S2). We previously reported that the A20/HSP70 signaling pathway is specifically activated in TNBCs^14^. Myc expression was determined to be the main target for clinical evaluation in currently ongoing Phase II trial^24^. Therefore, we examined the activity of Minnelide in targeting A20/HSP70 and Vimentin in MDA-MB231, 4T1, and EMT6 cell lines. TGFβ is a well-established factor for driving EMT and CSC phenotypes^33^ and is highly expressed by the immunosuppressive myeloid cell population^34^. Minnelide suppressed TGFβ-induced HSP70 and A20 expression, in addition to inhibiting the expression of the mesenchymal marker, vimentin (Fig. 4A and B). Next, we evaluated the effect of Minnelide on Myc and Brd4 expression. The latter is required for Myc expression^35^. Although they were not responsive to TGFβ stimulation in either cell line, Minnelide effectively inhibited Myc and Brd4 expression (Fig. 4A and B). In contrast, the EMT6 cell line was poorly responsive to TGFβ stimulation, and neither the expression levels of the indicated proteins were significantly changed or had notable Minnelide effect (Fig. 4C). Interestingly, Myc was inhibited at higher doses in EMT6 cells (Fig. 4C). Our study suggests that Myc is one of the main targets of Minnelide. Unprocessed raw data for each western blot analysis are provided in Supplementary Fig. S3A-C.

### Myc expression is higher in basal-like/ER-negative breast tumors and predict poor overall survival

Myc is one of the most frequently activated oncogenes and central drivers in multiple cancers, including breast cancer^36^. Although Myc alterations include frequent amplification, overexpression and rare mutations, it has been shown that high mRNA expression, not amplification, predicts poor overall survival in patients with breast cancer^37^. Furthermore, a renewed interest in targeting Myc with new-generation inhibitors is under preclinical development^38 39^ as well as in phase I clinical trials^40^. Using TCGA data set, we showed that Myc alterations were substantially higher in basal-like (59%) and ER-negative (48%) than in ER-positive (20%) breast cancers (Fig. 4D). As expected, elevated *Myc* mRNA expression predicted poorer overall survival in women with basal-like/ER-negative breast cancer (Fig. 4E and F). However, this was not predictive in patients with ER-positive tumors (Fig. 4G). Altogether these data confirmed the significance of Myc protein in basal-like/ER-negative tumors.

### Minnelide in combination with cyclophosphamide suppress tumor growth and eliminate metastasis by targeting CSCs and enhancing cytotoxic T cell infiltration

**Figure 5:**
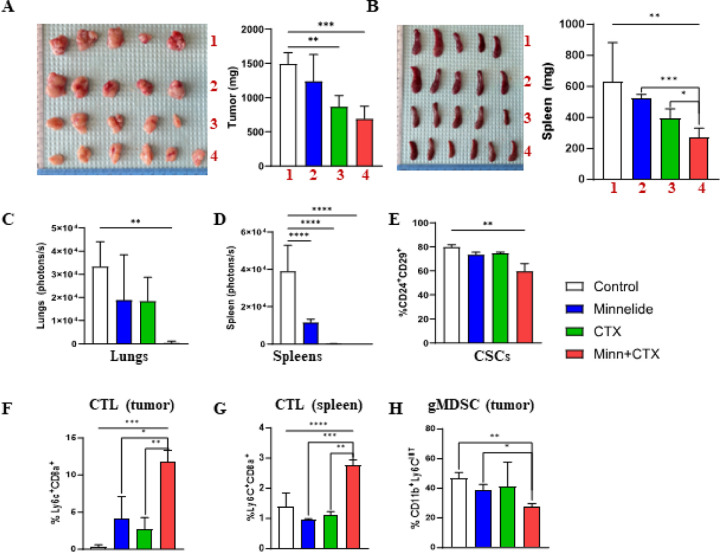
Minnelide in combination with CTX significantly reduces tumor growth and metastasis by reducing gMDSCs and enhancing T cell infiltration in 4T1 tumor bearing mice. Mice bearing 4T1 tumors were treated with single minnelide (n=5, 0.5mg/kg, daily), single CTX (n=5, 150mg/kg, weekly) and in combination (n=6) for 5 weeks compared to controls (n=5). **A,** Size of primary tumors and their weights at the end point of the experiment. **B,** Size of matching spleens their weights at the end point of the experiment. **C,** Bioluminescent imagings of lungs from each treatment group compared to controls. **D,** bioluminescent imaging of spleens from each treatment group compared to controls. **E,** Reduced cancer stemness in the tumors of combination group measured by flow cytometry (n=3 per group). **F,** Increased tumor infiltrating CTLs (CD8a+Ly6C+) in the tumors of combination group determined by flow cytometry (n=3 per group). **G,** increased systemic CTLs from the spleens of combination group (n=3 per group) **H,** Low gMDSC (CD11b+Ly6Cint) levels in the tumors of combination group(n=5). Values were shown in percentages (± SD). One-Way ANOVA is used to compare test groups to control and t-test was applied between the conditions. *P < 0.05, **P < 0.01, ***P < 0.001.

The murine 4T1 tumor model is a well-established TNBC subtype that generates spontaneous metastasis in the lungs and other tissues by inducing an immunosuppressive pre-metastatic niche^30 41^. To evaluate the *in vivo* activity of Minnelide, we treated 4T1 tumor-bearing mice with Minnelide (0.5mg/kg daily), or CTX (150mg/kg weekly), or a combination of both drugs for 5 week. Although Minnelide alone had a modest activity in reducing tumor growth in 4T1 tumor-bearing mice, when combined with CTX, it significantly reduced tumor growth, compared to the single Minnelide or CTX treatments (Fig. 5A). As expected, reduced tumor growth was concordant with reduced spleen size in the respective animals (Fig. 5B). Next, we determined the impact of the combination therapy on spontaneous metastasis in the treated mice. Although single Minnelide or CTX modestly reduced spontaneous metastasis, combination of the two eliminated metastasis in the lungs and spleens as ex-vivo images by bioluminescence showed no signals (Fig. 5C and D). When analyzing residual tumors, we found that the CSC population was significantly reduced by the combination of Minnelide and CTX (Fig. 5E), suggesting it’s *in vivo* activity on tumor cells. We previously reported that cytotoxic T cells (CTL) were characterized by CD8^+^Ly6C^+^ phenotype in BALB/c mice^31^ and; therefore, we analyzed immune cells in residual tumors and spleens from treated mice. There was significantly higher CTL infiltration in residual tumors and spleens that were treated with a combination of Minnelide and CTX (Fig. 5F and G). The granulocytic subset of myeloid derived suppressor cell (gMDSC) population, defined by the CD11b^+^Ly6C^int^ phenotype, was also reduced in mice treated with combination therapy (Fig. 5H).

### Neoadjuvant combination therapy with Minnelide plus cyclophosphamide eliminates residual 4T1 tumors in syngeneic mice

**Figure 6:**
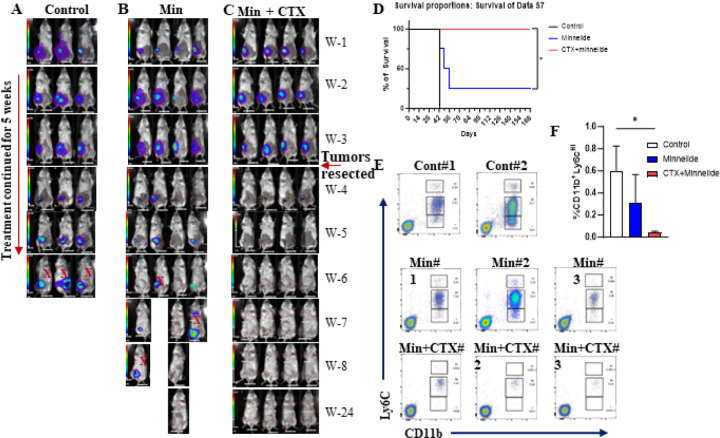
Minnelide in combination with CTX eliminates residual 4T1 tumors following surgery in syngeneic mouse model. Mice bearing 4T1 tumors were treated with minnelide (0.5mg/kg) daily for 3 weeks and primary tumors were resected, and treatment continued another 2 weeks. **A-C.** Minnelide alone significantly reduced the relapse after surgery, and it eliminated all residual tumors when combined with CTX. **D. S**urvival rates of 4T1 tumor bearing mice before and after the resections. **E.** Staining for Ly6C^HI^ and Ly6C^INT^ among CD11b+ myeloid cells from circulating MDSCs from control (n=2), single Minnelide (n=3) and combination (n=3) groups were measure by flow cytometry one week after the resection surgery. **F.** Bar graphs for immunosuppressive MDSCs showing lowering effects in combination group compared to control and Minnelide. Values were shown in percentages (± SD). One-Way ANOVA is used to compare test groups to control. *P < 0.05.

Our lab previously demonstrated in a 4T1 tumor model that mice show local and distant recurrences following the resection of primary tumors^31 41^. Consistent with our data (Fig. 5A and B), it has been widely reported that standard of care chemotherapeutics, including CTX, show modest activity in eliminating disseminated 4T1 tumor cells^42^. Therefore, we investigated the therapeutic potential of Minnelide plus CTX in a neoadjuvant setting to target disseminated 4T1 tumor cells. We treated 4T1 tumor-bearing mice with Minnelide (0.5mg/kg/daily) alone or in combination with CTX for 2 weeks in neoadjuvant setting before resecting the residual tumors, and then continued the treatment for another 3 weeks. Control mice developed local and distant recurrences within three weeks post-resection and were sacrificed (Fig. 6A). Minnelide alone modestly reduced relapse after surgery, and one mouse completely cleared residual tumors (Fig. 6B). In contrast, the combination of Minnelide and CTX therapy eliminated residual tumors in all mice that were free of local and distant recurrences for up to 6 months (Fig. 6C). We next evaluated circulating MDSCs in control and treated mice one-week after resection. Peripheral blood mononuclear cells (PBMCs) from mice treated with Minnelide plus cyclophosphamide contained substantially lower levels of monocytic and granulocytic MDSCs than those from Minnelide treated or control mice (Fig. 6D–F).

### Minnelide induces a distinct polarization of monocytes towards CD11c^+^CD86^+^ phenotype

**Figure 7:**
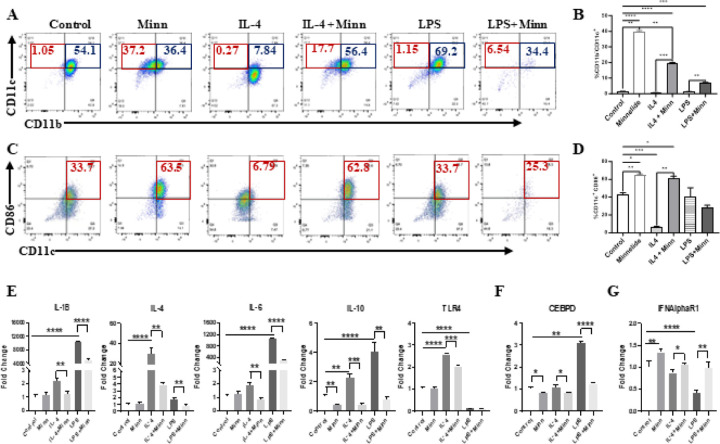
Minnelide induces monocyte-driven CD11c expansion in monocyte/macrophage-like cells *in vitro*. Raw264.7 cells were treated with IL-4 (10ng/ml), LPS (500ng/ml) in the presence or absence of Minnelide (100 uM) for 96 hours *in vitro*. **A.** Staining for CD11c and CD11b among live cells from 4 days of culture with indicated differentiating factors were measure by flow cytometer. Experiments were done in triplicates. **B.** Bar graphs for monocyte-driven differentiation to CD11c+ cells among CD11b- cells were increased by Minnelide. One-Way ANOVA applied for values compared to control and t-test was applied between the conditions. *P < 0.05, **P < 0.01, ***P < 0.001, ****P < 0.0001. **C.** Staining for CD11c and CD86 among live cells from 4 days of culture with indicated differentiating factors were measured by flow cytometer. Experiments were done in triplicates. **D.** Bar graphs for CD11c+CD86+ cells were increased by Minnelide. **E.** Expressions of immune activates genes on differentiated monocytes. Experiments were done in 2 independent times in triplicate and values were shown in log scale (± SD). One-Way ANOVA is used to compare test groups to control and t-test was applied between the conditions. *P < 0.05, **P < 0.01, ***P < 0.001, ****P < 0.0001.

To further examine the effect of Minnelide on myeloid cell population, we utilized monocyte/macrophage cell line, RAW264.7 (called RAW4 hereafter) which is widely used to induce macrophage polarization in response to various factors including IL-4, IL-13 and LPS^43 44^. RAW4 cells, under normal culture conditions, are mainly CD11b positive (> 95%) and roughly half of these (54%) express both CD11b and CD11c surface markers (CD11b^+^CD11c^+^), while small fraction of cells (~ 1%) are characterized by single CD11c^+^ phenotype (Fig. 7A). As previously reported, IL-4 effectively polarize these populations towards single Cd11b^+^ phenotype (~ 87%) reducing the CD11b^+^CD11c^+^ phenotype from 54.1–7.84% whereas LPS increased the CD11b^+^CD11c^+^ population from 54.1–69.2% (Fig. 7A blue boxes). Consistent with the literature, IL-4 enriched CD11b^+^Ly6G^+^ subset which were effectively depleted by Minnelide (Supplementary Fig. 4A and B). In addition, Minnelide treatment not only effectively polarized the RAW4 cells towards single CD11c^+^ phenotype (37.2%) compared to the control (1.05%), but it also reversed the effect of IL-4 or LPS treatment increasing the CD11c^+^ subset from 0.27–17.7% and 1.15–6.54% respectively (Fig. 7A red boxes and B). TCGA data analyses showed that higher CD11c (ITGAX) expression predicted better survival among patients with breast cancer (Supplementary Fig. S5). Because the CD86 surface marker is upregulated during dendritic cell maturation and type I macrophage polarization^45 46^, we examined whether Minnelide could expand this CD86 + subset in RAW4 cells. Although CD11b^+^CD86^+^ population was reduced (data not shown), there was a substantial expansion of the CD11c^+^CD86^+^ subset (from 33.7–63.5%) upon Minnelide treatment compared to that in the control (Fig. 7C red boxes and D). As expected, IL-4 treatment significantly reduced this CD11c^+^CD86^+^ subset to 6.79% from 33.7% in the control whereas LPS had no effect (Fig. 7C red boxes and D). When treated in combination, Minnelide reversed the effect of IL-4 on the CD11c^+^CD86^+^s phenotype increasing it to 62.8% from 6.79% in the single IL-4 treatment (Fig. 7C red boxes and D). Furthermore, Minnelide was able to reverse IL-4 or LPS-induced expression of cytokines, *IL-1B, IL-4, IL-6, IL-10* and *TLR4* which drive the polarization of myeloid cells towards immunosuppressive macrophages and MDSCs (Fig. 7E). Consistent with this notion, the transcription factor, CCAAT/enhancer-binding protein delta (CEBPδ) involved in macrophage differentiation^47^, was also suppressed by Minnelide (Fig. 7F). Interestingly, Minnelide induced the upregulation of type I interferon receptor (*IFNAR1*), which has been shown to restrict the acquisition of immunosuppressive activity in myeloid progenitors^48^, which is in line with its downregulation by IL-4 or LPS (Fig. 7G).

## Discussion

The standard of care chemotherapeutics remains the mainstream treatment for patients with the basal/TNBC subtype^1^ despite the early clinical development of molecularly targeted therapeutics^49^. Although cytotoxic agents have shown significant benefits in the neo-adjuvant setting and in extending the life of patients, the majority of those relapse and develop more aggressive disease. Similarly, although 4T1 tumors, classified as murine TNBC^30^, respond to neoadjuvant CTX treatment in syngeneic mice by significantly reducing tumor size, the standard of care chemotherapeutics including CTX fail to eliminate disseminated tumor cells^42^. The aggressive properties of basal/TNBC subtype are attributed to their heterogeneity and phenotypic (EMT/CSC) plasticity^32^ as well as their ability to drive an immunosuppressive tumor microenvironment^6^. Therefore, therapeutics designed to target both tumor intrinsic pathways and TME are expected to improve disease outcome in patients with the basal/TNBC subtype.

Minnelide, has shown promising preclinical activity against multiple malignancies^7 8 23^, is currently being evaluated in Phase II clinical trial (NCT04896073) for patients with advanced refractory pancreatic carcinoma^24^. Therefore, we evaluated the activity of Minnelide in a series of human and murine breast cancer cell lines and demonstrated that Minnelide induces dose dependent apoptotic cell death specifically in basal/TNBC cell lines. Because Minnelide targets transcriptional super enhancers (BRD4), Myc and HSP70, which are all implicated in cancer stemness of basal/TNBC^10 11 14 28^, we reasoned that it may target CSC subsets in these cell lines. Minnelide effectively depleted CSC population in human (MDA-MB231 and SUM159) and murine TNBC (4T1) cell lines, while having no significant effect on CSC from luminal MCF7 or murine EMT6 cell lines. Consistent with our findings, Minnelide has been shown to target CD133 + tumor-initiating cells in pancreatic ductal adenocarcinoma^12^. The anti-tumorigenic activity of Minnelide is attributed to its unique ability to target multiple oncogenic signaling molecules, including the HSP70 signaling pathway, in multiple malignancies^13^. Moreover, we previously demonstrated that upregulation of the A20/HSP70 pathway expanded the CSC population in the TNBC subtype in response to TNFα^14^. As expected, Minnelide mediated depletion of CSCs in TNBC may be mediated by targeting the A20/HSP70 signaling axis.

Minnelide binds to the XBP subunit of the transcription factor II H (TFIIH)-BRD4 super-enhancer complex that regulates many targets, including *c-Myc* expression^9^. It was also reported that the anti-tumor activity of BRD4 inhibitor in TNBC was shown to be mediated by the downregulation of Myc expression^50^. Thus, the scientific exploratory end points included the evaluation of *Myc* expression and accessibility of loci for the *Myc* gene in pre- and post-treatment tumors^24^. Although Myc was not induced by TGFβ, Minnelide most effectively inhibited Myc expression in both MDA-MB231 and 4T1 TNBC cell lines, and moderately downregulated Myc expression in EMT6 cells. This is significant because Myc is widely implicated oncogene in approximately 70% of malignancies and play a role in therapeutic resistance^50^. Furthermore, Myc gene alterations and overexpression were significantly higher in Basal-like and ER-negative breast cancer subtypes than the luminal subtype. Although, it was considered “undraggable” until recently^51^, new generation small molecule inhibitors of Myc are in preclinical development and early clinical trials^40^. Consistent with our data, Myc inhibition also depleted CSC populations in the TNBC subtype^52^. To the best of our knowledge, this is the first study to demonstrate significant inhibition of Myc by Minnelide in TNBC subtype. Preclinical studies and ongoing clinical trials suggest that Minnelide sensitizes cancer cells to conventional chemotherapy^8 20 23^. In line with this evidence, we demonstrated that although it showed a modest in vivo activity, it effectively sensitized 4T1 tumors to cyclophosphamide. Increased tumor infiltration and systemic expansion of cytotoxic T cells in mice treated with combination therapy also indicated that Minnelide may reprogram the immunosuppressive TME. This is supported by a significant reduction in gMDSCs, which we previously demonstrated to drive pulmonary metastasis in 4T1 tumor-bearing mice^32^. We and others have reported that 4T1 tumors quickly relapse after surgical resection of primary tumors due to disseminated tumor cells^32^ and conventional chemotherapeutics fail to eliminate these disseminated tumor cells^42^. Women with TNBC show a pathological complete response to platinum based agents in the neoadjuvant setting; however, high residual disease burden post-surgery is correlated with a higher risk of recurrence and death^53^. Surprisingly, the combination of Minnelide with cyclophosphamide effectively eliminated these residual tumors following surgery and mice were free of local and metastatic recurrences for up to 6-months. Complete elimination of gMDSCs in mice treated with Minnelide in combination with cyclophosphamide suggested reprogramming of the microenvironment towards anti-tumorigenic immunity. This is consistent with a previous report that Minnelide targets pro-tumorigenic stroma, a hallmark of pancreatic carcinoma^20^. Despite the overwhelming evidence of clinical significance, targeting or reprogramming immunosuppressive macrophages/MDSCs has been challenging, in part due to their phenotypic and functional heterogeneity^46^. Among the cytokines, IL-4 has been well characterized to polarize myeloid cells towards type II macrophages/MDSCs. Owing to its significance, the therapeutic utility of targeting IL-4Rα has been explored in preclinical and early clinical trials. It was recently shown that IL-4Rα targeting antibody, dupilumab effectively reduced circulating monocytes and expanded tumor-infiltrating CD8 T cells^43^. Minnelide effectively reversed the IL-4 induced phenotypic polarization of RAW4 cells towards CD11c^+^CD86^+^ phenotype which is primarily expressed by mature dendritic cells (DCs). Therefore, we postulate that DCs with CD11c^+^CD86^+^ phenotype within the tumor microenvironment may function as antigen-presenting cells, driving the infiltration and activation of cytotoxic T cells. This is supported by a previous study suggesting that DCs with CD11c^+^CD86^+^ phenotype are capable of migrating to tumor-draining lymph nodes for proper antigen presentation^45^. Moreover, the CD86 marker is also expressed during anti-tumorigenic type I macrophage polarization^46^.

In conclusion, we provide ample evidence that Minnelide targets tumor intrinsic pathways while reprogramming the immunosuppressive microenvironment and enhancing T cell infiltration in syngeneic mice. Our findings provide significant promise for its clinical utility and thus warrant further investigation in clinical settings.
